# X-linked recessive ichthyosis with X-linked retinoschisis in two brothers: a case report

**DOI:** 10.3389/fgene.2026.1781409

**Published:** 2026-07-20

**Authors:** Kou Liu, Xiaojun Du, Xiaohan Yang, Chunli Chen

**Affiliations:** 1 Beijing Tongren Eye Center, Beijing Tongren Hospital, Capital Medical University, Beijing, China; 2 Tianjin Eye Institute, Tianjin Eye Hospital, Tianjin Medical University, Tianjin, China; 3 Department of Ophthalmology, Shengli Oilfield Central Hospital, Dongying, China

**Keywords:** case report, RS1, STS, X-linked ichthyosis, X-linked retinoschisis

## Abstract

**Background:**

X-linked ichthyosis (XLI) and X-linked retinoschisis (XLRS) are both inherited in an X-linked recessive manner. To date, no prior reports document both conditions’ simultaneous occurrence. This case presents two brothers who were diagnosed with XLI combined with XLRS in a family.

**Case presentation:**

An 11-year-old boy (Case 1) presented to our hospital due to decreased bilateral vision detected during a physical examination over the past month. One month after birth, the parents noticed the child’s skin was dry and rough. He was diagnosed with “ichthyosis” at another hospital, and after treatment, the condition improved. His mother had also been previously diagnosed with ichthyosis. The child was a full-term infant delivered by cesarean section, and his parents were not closely related. There was no family history of eye disease. Physical examination showed dry skin with diamond-shaped scales. The best-corrected visual acuity was 0.40 logMAR in both eyes. No abnormalities were observed in the anterior segments of both eyes. Fundus examination revealed petaloid macular edema, and OCT showed numerous cystic changes in the macular area of both eyes. His 6-year-old younger brother (Case 2) exhibited similar systemic and ocular features. Comprehensive ophthalmic and genetic examinations were performed on the entire family, revealing that both children carried an RS1 gene mutation, with the mutation site at c.545G>T/p.Arg182Leu, inherited from their mother. Additionally, a deletion variant of approximately 478 kb was found at the X chromosome p22.31 location, completely covering the STS gene region. Based on the genetic testing and ocular examination, both children were finally diagnosed with bilateral XLRS and XLI.

**Conclusion:**

This case expands the mutation spectrum of XLRS in Chinese patients and, for the first time, reports the ocular and systemic manifestations when two different disease genes, STS and RS1, coexist. The family members vividly demonstrate the phenotypic and genotypic individual heterogeneity associated with hereditary eye diseases. A comprehensive analysis of clinical phenotype and genotype improves clinical diagnosis and genetic testing accuracy, providing a clinical approach for a more comprehensive understanding of the disease.

## Introduction

Ichthyosis is a genetic keratinization disorder characterized by dry skin and fish-scale-like scales. X-linked ichthyosis (XLI) is a common subtype of ichthyosis, typically presenting in neonates or within the first year of life (Gutiérrez-Cerrajero et al., 2023). It is caused by a deficiency of steroid sulfatase (STS), the incidence rate is 1/6000 to 1/2500, almost exclusively seen in males (Crane and Paller, 2024).

X-linked retinoschisis (XLRS) is an inherited retinal disorder associated with splitting of the neurosensory retina (Ku et al., 2023). XLRS is caused by mutations in the RS1 gene and follows an X-linked recessive inheritance pattern. Its incidence is estimated at 1:25,000 to 1:5,000, and it typically affects both eyes, primarily males in childhood and adolescence. Visual acuity typically deteriorates during the first and second decades of life, followed by a relatively stable period until the fifth and sixth decades, with possible further decline in later life due to macular atrophy. Some patients may present in early infancy with strabismus or nystagmus (De Silva et al., 2021). We performed genetic analysis on two children with XLI with XLRS, as well as their family members, to identify the potential pathogenic causes.

## Case presentation

### Case 1

An 11-year-old boy presented to our hospital in January 2024 after reduced vision was detected during a routine school screening. Past medical history revealed that the patient’s parents had noticed dry and rough skin 1 month after his birth. He was diagnosed with “ichthyosis” in the dermatology department of another hospital and treated with topical tretinoin, with subsequent improvement. Family history revealed that the patient’s mother had also been diagnosed with ichthyosis, although her symptoms were mild. Both parents underwent ophthalmic examinations, including fundus photography, OCT and fundus autofluorescence imaging, with no abnormalities observed. The child was born at full term via cesarean section and was the first child of non-consanguineous parents.

**FIGURE 1:**
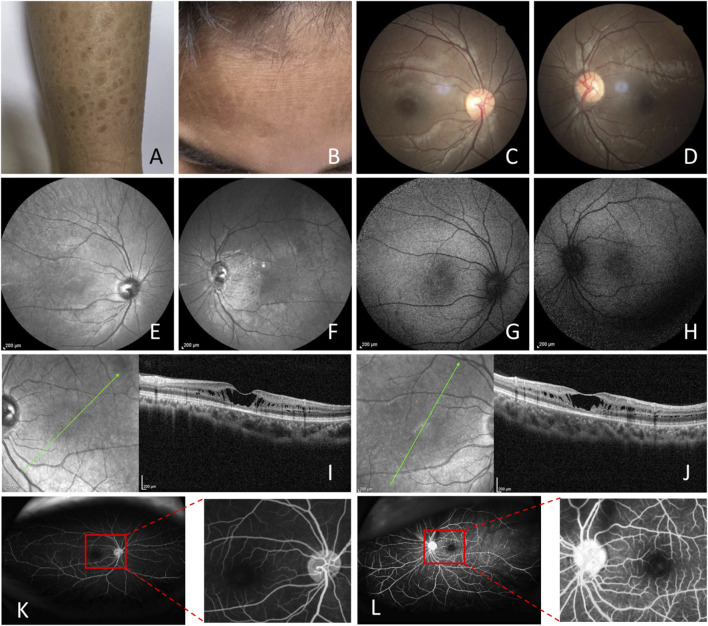
Case 1 - Skin and Ocular Examinations. **(A,B)** Rough skin on the legs and forehead with “fish-scale” changes. **(C,D)** Fundus examination shows petal-like retinal cysts in the macular region of both eyes. **(E,F)** Fundus infrared imaging reveals no significant abnormalities in the macular region of either eye. **(G,H)** Autofluorescence imaging shows mixed high and low fluorescence in the macular area. **(I,J)** OCT reveals retinal schisis-like edema in the outer nuclear layer of both eyes, with damage to the outer/inner segments of photoreceptors. **(K)** Right eye FFA shows punctate hyperfluorescence in the macula with no significant leakage. **(L)** Left eye FFA shows high fluorescence in the optic disc, and punctate hyperfluorescence in the macula with no significant leakage.

General physical examination revealed dry skin over the entire body, with rhomboid or polygonal brown scales of varying sizes on the limbs and forehead (Figures 1A,B). Ophthalmic examination showed the best-corrected visual acuity (BCVA) was 0.40 logMAR in both eyes. The intraocular pressure was 12 mmHg in both eyes, and slit-lamp examination revealed no significant abnormalities in the anterior segments. Fundus examination revealed a spoke-wheel pattern in the macular region, suggestive of foveal schisis (Figures 1C,D). No peripheral retinoschisis was identified. In addition, no other retinal abnormalities, including retinal flecks, vascular attenuation, retinal detachment, or vitreous hemorrhage, were observed. Fundus infrared scanning showed no significant abnormalities in either eye (Figures 1E,F). Autofluorescence imaging revealed mixed hyper- and hypo-autofluorescence in the macular region of both eyes (Figures 1G,H), consistent with foveal schisis characterized by radially arranged schitic cavities producing alternating autofluorescence signals. OCT revealed a large cyst in the outer nuclear layer of the macular area in both eyes, and numerous small cysts connected by bridge-like structures near the macula (Figures 1I,J). Disruption of the ellipsoid zone and atrophy of the photoreceptor layer were observed in the macular region. Fundus fluorescein angiography showed punctate hyperfluorescence in the macular area of both eyes, without leakage. Hyperfluorescence of the left optic disc was also observed, possibly related to fluorescein accumulation within small cavities in the peripapillary nerve fiber layer (Figures 1K,L).

### Case 2

A 6-year-old male, and the younger brother of Case 1, presented for an eye examination due to his older brother’s poor vision. Past medical history revealed that the parents had noticed that the child’s skin was dry 2 months after birth. He was diagnosed with “ichthyosis” and treated with topical tretinoin. Family history revealed that his older brother and mother also suffered from ichthyosis. The parents had no history of ocular diseases. The child was born at full term via cesarean section.

**FIGURE 2:**
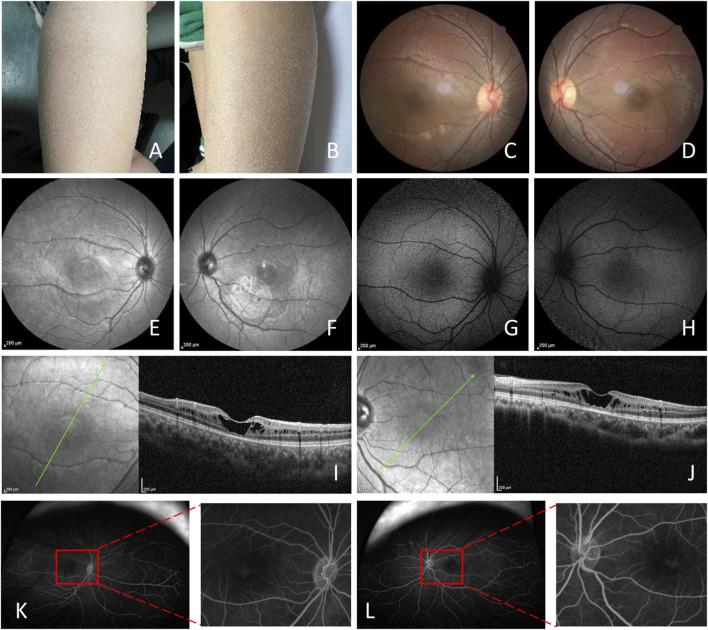
Case 2 - Skin and Ocular Examinations. **(A,B)** Dry and rough skin on the limbs. **(C,D)** Fundus examination shows spoke-wheel-like changes in the macular region of both eyes. **(E,F)** Fundus infrared imaging reveals schisis-like changes in the macula of both eyes. **(G,H)** Autofluorescence imaging shows a small amount of hyperfluorescence. **(I,J)** OCT reveals retinal schisis in both eyes, with photoreceptor layer damage. **(K)** Right eye FFA shows punctate hyperfluorescence in the macular region with no significant leakage. **(L)** Left eye FFA shows punctate and patchy hyperfluorescence in the macular region with no significant leakage.

General physical examination revealed dry and rough skin, particularly on the limbs, with a milder condition compared to his older brother (Figures 2A,B). Ophthalmic examination results were similar to those of his older brother. BCVA was 0.40 logMAR in both eyes, intraocular pressure was 13 mmHg, and slit-lamp examination revealed no abnormalities in the anterior segments. Fundus examination revealed spoke-wheel-like cystoid macular edema in both eyes, without peripheral retinoschisis or other retinal abnormalities (Figures 2C,D). Fundus infrared imaging showed spoke-wheel-like changes in the macular region of both eyes (Figures 2E,F). Fundus autofluorescence imaging revealed punctate hyperautofluorescence in the macular region (Figures 2G,H). OCT revealed schisis-like retinal edema in the outer nuclear layer of both eyes, with photoreceptor layer defects (Figures 2I,J). FFA showed punctate and patchy hyperfluorescence in the macular region without leakage, and no significant differences between the two eyes (Figures 2K,L).

Based on the clinical manifestations of the two patients and their mother, X-linked retinoschisis (XLRS) combined with X-linked ichthyosis (XLI) is highly suspected. After obtaining informed consent from the parents, peripheral blood samples were collected from the patients and their parents for high-throughput sequencing and genetic testing. All subjects underwent Sanger validation (Beijing Novogene Technology Co., Ltd.). Genetic variant pathogenicity assessment for novel variants was conducted according to the 2015 guidelines for sequence variant interpretation issued by the American College of Medical Genetics and Genomics (ACMG).

**FIGURE 3:**
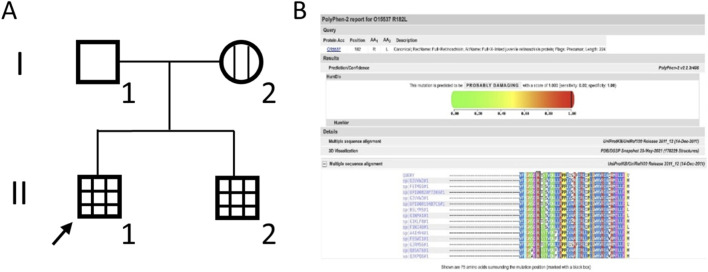
The pedigree chart and genetic testing. **(A)** 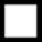: Normal male; 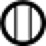: Female with XLI; 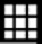: Male with both XLI and XLRS. **(B)** The mutation site (c.545G>T/p.Arg182Leu) has a high pathogenic rate.

The inheritance pattern in this family is X-linked recessive. Complete family information was collected, and a pedigree was drawn (Figure 3A). The results showed that both affected children carried the RS1 gene variant, with the mutation located at c.545G>T/p.Arg182Leu, which is a missense mutation. Analysis revealed that the mutation was inherited from the mother, while the father was wild-type. This mutation was not detected in the gnomAD East Asian population or the 1000 Genomes Project Chinese population. According to the ACMG criteria, the pathogenicity of this variant is currently classified as uncertain. However, the phenotypes of the variant carriers and the family history are highly consistent with XLRS. The variant site was predicted to be “probably damaging” by the PolyPhen-2 software, and the amino acid site is highly conserved across multiple species, suggesting a high likelihood of pathogenicity (Figure 3B). Whole exome sequencing with CNV analysis revealed a deletion variant approximately 478 kb in length at the X chromosome p22.31 region in the proband (Case 1) (Figure 3B). Database searches showed that this chromosomal region fully covers the STS gene (chrX:7065293-7272684), and a deletion in this region can lead to X-linked recessive ichthyosis. Based on the patient’s clinical presentation and according to the ACMG variant classification guidelines (PMID:25741868, 31690835), the chromosomal deletion variant was classified as pathogenic. ERG was not performed in this patient, which represents a limitation of this report. In conclusion, based on the clinical findings and genetic test results, the two patients were ultimately diagnosed with X-linked retinoschisis and X-linked ichthyosis. The patients were advised to use low vision aids and to undergo regular follow-up. What’s more, they received topical tretinoin therapy for dry skin.

## Discussion

X-linked ichthyosis (XLI) is primarily characterized by generalized skin dryness, with the appearance of scaly, polygonal, and brownish patches, mainly affecting the extensor surfaces of the limbs or the trunk. It often manifests within a few weeks after birth. In 1978, Shapiro et al. confirmed that the STS gene is the causative gene for XLI (Shapiro et al., 1978). Approximately 90% of XLI cases are associated with a deletion of the Xp22.31 region, which includes the STS gene, while a minority involve small fragment deletions or point mutations (Cañueto et al., 2010). Whole exome sequencing with CNV analysis of the proband revealed a 478 kb deletion variant at the X chromosome p22.31 region, which fully covers the STS gene region. This chromosomal variant has not been reported in the Human Genome Polymorphism Database (DGV_GS), and this variant leading to XLI is a novel finding. The STS gene is a key gene involved in the recycling of cholesterol. When the STS gene is deleted, the structure and function of the encoded protein are altered, which in turn impairs the degradation of certain cholesterol sulfate esters, leading to an accumulation of cholesterol sulfate and a reduction in its degradation products in the local area. This imbalance disrupts the normal shedding and integrity of the stratum corneum, resulting in clinical symptoms such as scaling and impaired skin barrier function (Shapiro et al., 1978). However, there is currently no effective cure for ichthyosis, and topical emollients and oral retinoid therapy are the primary treatments for XLI.

Ichthyosis patients may have other systemic complications in addition to skin manifestations. It has been reported that about 20% of XLI male patients have cryptorchidism. Moreover, the deletion at the Xp22.31 region is considered a susceptibility factor for neurodevelopmental disorders in males. Boys with XLI may exhibit symptoms such as autism, intellectual disability, epilepsy, and developmental delay. Some XLI patients may also have eye diseases, mainly including ectropion, corneal opacity, cataract, and retinitis pigmentosa (Diaz et al., 2013). Any genomic imbalance, deletion, or duplication of the X chromosome usually has a more severe impact on males than on females because males have only one X chromosome and lack the gene redundancy of females. In males, most cytogenetically visible X chromosome deletions involve the terminal part of Xp (Xp22.2→Xpter). These deletions are considered one of the causes of contiguous gene syndromes. The phenotypes vary depending on the extent and location of the deletion, resulting in a diverse range of clinical manifestations such as ichthyosis (caused by STS gene abnormalities), Leri-Weill dyschondrosteosis (caused by SHOX gene alterations), punctate chondrodysplasia (caused by CDPX1 gene abnormalities), intellectual disability (caused by NLGN4 gene abnormalities), Kallmann syndrome (caused by KAL1 gene abnormalities), and ocular albinism (caused by GPR143 gene abnormalities). Through a literature review, we have compiled case reports of X chromosome deletions involving STS abnormalities along with other gene abnormalities (Table 1). These cases further confirm the association between X chromosome gene abnormalities and a variety of clinical phenotypes.

**TABLE 1:** Other genotypes and phenotypes with STS abnormalities.

Author	Year	Variant gene 1	Disease	Variant gene 2	Disease
Van Esch et al. (2005)	2005	STS	XLI	VCX-A	Intellectual disability
Kent et al. (2008)	2008	STS	XLI	NIGN4	Attention deficit hyperactivity disorder (ADHD), autism
Ramesh et al. (2011)	2009	STS	XLI	FLG	Ichthyosis vulgaris
Hernández-Martín et al. (2010)	2010	STS	XLI	COL7A1	Nutritional epidermolysis bullosa (DEB)
Preumont et al. (2010)	2010	STS	XLI	HDHD1	Inability to encode pseudouridine-5′-phosphatase
Pallagatti et al. (2012)	2012	STS	XLI	COL7A1	Nutritional epidermolysis bullosa (DEB)
Cho et al. (2012)	2012	STS	XLI	OA1	Oculocutaneous albinism
Ben Khelifa et al. (2013)	2013	STS	XLI	VCX3A	Mental retardation (MR)
Xu et al. (2015)	2015	STS	XLI	ANOS1 (KAL1)	Kallmann syndrome
Bai et al. (2016)	2016	STS	XLI	UGT1A1	Crigler-Najjar syndrome (CN-I)
Abdel-Hamid et al. (2016)	2016	STS	XLI	ASPM	Microcephaly
Ohyama et al. (2019)	2018	STS	XLI	SHOX	Short stature, decreased bone density, epilepsy, cryptorchidism
Wang et al. (2018)	2018	STS	XLI	FLG	Ichthyosis vulgaris
Ma et al. (2020)	2020	STS	XLI	ANOS1 (KAL1)	Kallmann syndrome, obesity, strabismus
Sait et al. (2021)	2021	STS	XLI	ANOS1, SHOX, ARSE	Kallmann syndrome

XLRS is an X-linked recessive disorder affecting male children, often detected during routine vision screening or due to difficulties in the classroom. The disease usually involves both eyes, may be asymmetric, and can present in infancy with nystagmus or strabismus. Fundus findings include foveal schisis and cystic macular changes with a spoke-wheel or petal-like pattern. Visual acuity typically declines during the first two decades of life, stabilizes thereafter, but may worsen in later life due to macular atrophy (Ku et al., 2023).

Differential diagnoses for XLRS include several hereditary and acquired retinal disorders. Unlike XLRS, retinal detachment typically shows separation of the neurosensory retina from the retinal pigment epithelium on OCT rather than intraretinal schisis cavities with bridging structures (Fahim et al., 2017). Acquired or degenerative retinoschisis usually occurs in older individuals and mainly involves the peripheral retina, whereas XLRS typically presents in male children with characteristic foveal schisis. Goldmann-Favre vitreoretinal degeneration, an NR2E3-associated retinal dystrophy, may show schisis-like changes but is usually accompanied by progressive night blindness and characteristic ERG abnormalities (Herrador-Montiel et al., 2012). Retinitis pigmentosa is mainly characterized by early night blindness, peripheral bone-spicule pigmentation, attenuated retinal vessels, and progressive photoreceptor dysfunction, features that were not observed in our cases.

Variations in the RS1 gene are the main cause of XLRS. This gene is located at Xp22 and encodes a highly conserved extracellular protein, RS1 protein, also known as retinoschisin, which contains 224 amino acids. Retinoschisin is mainly expressed in photoreceptor and bipolar cells of the retina and plays an important role in maintaining retinal cell-cell adhesion and structural integrity (Ku et al., 2023). Mutations in the RS1 gene disrupt the function or secretion of retinoschisin, leading to impaired intercellular adhesion and subsequent splitting of the neurosensory retina. Different XLRS patients exhibit extensive heterogeneity in retinal structural and functional changes, and a clear genotype-phenotype correlation has yet to be established. In our report, genetic testing showed that both children carried the same RS1 gene mutation as their mother, with the mutation at c.545G>T/p.Arg182Leu.

Common complications of XLRS include rhegmatogenous or tractional retinal detachment (0%–22%), vitreous hemorrhage (4%–40%), retinal pigment epithelial changes (26%–29%), retinal vascular abnormalities with or without exudation (6%–11%), and neovascular glaucoma. These complications can further impair visual function, with a blindness rate exceeding 60% in adult patients (Wakabayashi et al., 2023). Currently, there is no curative treatment for XLRS, and clinical management mainly focuses on symptomatic treatment and managing complications. Carbonic anhydrase inhibitors can reduce intraretinal fluid accumulation and improve schisis cavities in some patients (Hensman et al., 2024). Laser photocoagulation is generally not recommended, as it may increase the risk of rhegmatogenous retinal detachment. In cases of severe complications, such as persistent vitreous hemorrhage or retinal detachment, surgical intervention, including pars plana vitrectomy, may be required (Das et al., 2025). Gene therapy, by restoring functional RS1 expression to reestablish retinal cell connections and reduce schisis cavity formation, has emerged as a promising approach for treating XLRS. In animal models, AAV-mediated RS1 gene replacement has been shown to improve retinal structure and enhance ERG responses. Early clinical trials indicate that intravitreal injection of AAV-RS1 can provide potential structural benefits with an acceptable safety profile. However, immune responses, limited viral transduction efficiency, and long-term efficacy remain to be addressed (Cukras et al., 2018). XLRS is usually unrelated to other eye diseases, but it has been reported that in NDP gene knockout mice, Norrie disease and XLRS have overlapping features. However, the molecular mechanisms between these two diseases are not yet clear.

## Conclusion

XLI and XLRS are both X chromosome recessive genetic diseases, and there have been no previous reports of co-occurrence. The association between their pathogenic mechanisms remains unclear. After a clear genetic diagnosis of the proband, precise genetic counseling and marriage and childbearing guidance can be provided, which is of great significance for the prevention of the birth of affected children. With the development of genetics and molecular biology, our understanding of the molecular genetics, pathogenesis, and disease progression of XLI and XLRS is constantly deepening, providing new directions for the diagnosis, intervention, and treatment of these diseases.

This case not only expands the mutation spectrum of XLRS in Chinese patients but also reports for the first time the ocular and systemic manifestations when the two different disease genes, STS and RS1, coexist. The comprehensive analysis of clinical phenotypes and genotypes helps to improve the level of clinical and genetic diagnosis and provides a clinical approach for a more comprehensive understanding of the diseases.

## Data Availability

The original contributions presented in the study are included in the article/supplementary material, further inquiries can be directed to the corresponding authors.
